# From Genotype to Therapeutic Monitoring: Enhancing Tamoxifen Efficacy in Breast Cancer Treatment

**DOI:** 10.1002/jcph.70103

**Published:** 2025-09-05

**Authors:** Ana Flávia Mendes Batista, Letícia Penteado Petrolli, Maria Paula Marques Pereira, Adriana Rocha, Jurandyr Moreira de Andrade, Vera Lucia Lanchote, João Paulo Bianchi Ximenez

**Affiliations:** ^1^ School of Pharmaceutical Sciences of Ribeirao Preto University of Sao Paulo Ribeirao Preto SP Brazil; ^2^ Ribeirao Preto Medical School University of Sao Paulo Ribeirao Preto SP Brazil

**Keywords:** CYP2D6, endoxifen, pharmacogenetics, pharmacokinetics, tamoxifen, therapeutic drug monitoring

## Abstract

Endoxifen is the most active metabolite of tamoxifen and plays a central role in its therapeutic efficacy. However, significant interindividual variability in endoxifen plasma concentrations, driven by both genetic and non‐genetic factors, may result in subtherapeutic exposure for a substantial subset of patients. This study evaluated the influence of CYP2D6 phenotype and age on endoxifen steady‐state concentrations and explored the clinical utility of therapeutic drug monitoring (TDM) to guide tamoxifen therapy. A total of 63 breast cancer patients receiving tamoxifen 20 mg daily for at least 3 months were enrolled. Patients were genotyped for CYP2D6 using TaqMan assays and classified as normal metabolizers (NMs, n = 49), intermediate metabolizers (IMs, n = 13), or ultrarapid metabolizers (UMs, n = 1). Plasma concentrations of tamoxifen and its metabolites were quantified by LC‐MS/MS at steady state. Endoxifen levels were significantly lower in IMs (7.13  ng/mL; 95% CI: 3.38‐15.08) compared to NMs (22.66  ng/mL; 95% CI: 18.57‐27.66; *P*  <  .001). Subtherapeutic endoxifen concentrations (<6  ng/mL) were observed in 23.1% of IMs and 4.1% of NMs. These results support the combined use of CYP2D6 genotyping and TDM as the optimal strategy for personalizing tamoxifen therapy and minimizing the risk of subtherapeutic endoxifen exposure.

## Introduction

Breast cancer is the most diagnosed cancer among women, and it is the second leading cause of cancer incidence worldwide, representing 11.6% of all cases.^1^ Tamoxifen is regularly used in the endocrine adjuvant treatment of women with estrogen receptor (ER)‐positive early breast cancer, reducing the risk of disease recurrence and mortality in these patients.^2^, ^3^

Tamoxifen has been used in breast cancer treatment since the late 1960s, with a dose of 20 mg/day of tamoxifen for at least 5 years.^4^ Tamoxifen is considered a prodrug, as it requires bioactivation by cytochrome P450 (CYP) enzymes for the formation of two active metabolites, endoxifen and 4‐hydroxy‐tamoxifen, which have 30‐ to 100‐fold more potent antiestrogenic effects than the parent drug.^5^ CYP3A4/5 first converts most of the tamoxifen to N‐desmethyltamoxifen, which is a weak selective ER modulator. N‐desmethyltamoxifen is subsequently converted by CYP2D6 to endoxifen. 4‐hydroxy‐tamoxifen is also converted from tamoxifen, mainly by CYP2C9 and by CYP2D6, 3A4, 2B6, and 2C19.^6^, ^7^

Given that plasma concentrations of endoxifen significantly exceed those of 4‐hydroxy‐tamoxifen, endoxifen is regarded as the primary contributor to the drug's clinical efficacy.^5^, ^8^ Furthermore, the endoxifen is characterized by a high interindividual variability, resulting in considerable interindividual differences in plasma levels among tamoxifen‐treated patients, with a substantial proportion presenting subtherapeutic concentrations and a high risk of breast cancer recurrence.^9^, ^10^ Genetic polymorphism of CYP2D6 is one of the most important factors involved in its high variability in pharmacokinetics and plasma concentration.^11^ For this reason, the Clinical Pharmacogenetics Implementation Consortium (CPIC) published in 2018^12^ a guideline with the purpose of providing clinical information to allow the interpretation of clinical CYP2D6 genotype tests, which could be used as a guide for tamoxifen prescription.

However, a large proportion of the variability in endoxifen levels remains unexplained, suggesting that multiple genetic and non‐genetic factors contribute in varying degrees.^11^, ^13^, ^14^ Several studies sought to demonstrate a threshold for the plasma concentration of endoxifen that would result in the expected effects. A threshold of 16  nM (5.97  ng/mL) has been reported by two studies, in which patients with plasma concentrations below that could be considered a risk group due to a higher association with recurrence and death.^9^, ^15^ Thus, therapeutic drug monitoring (TDM) has been a current suggestion to determine endoxifen plasma concentration in the steady state (C_SS_) to dose optimization during treatment with tamoxifen.^13^, ^14^, ^16^

For these reasons, this study raises the question of how much the CYP2D6 phenotype may influence the endoxifen steady‐state plasma concentration and what the clinical benefits of TDM during treatment may be.

## Methods

### Study Design

The study was approved by the institutional review board of the School of Pharmaceutical Sciences of Ribeirão Preto, University of São Paulo, São Paulo, Brazil, and of the General Hospital of Ribeirão Preto Medical School, University of São Paulo, São Paulo, Brazil (Record number: 35539714.7.0000.5403) and by the institutional review board of the Instituto Nacional de Câncer, Rio de Janeiro (Record number: 50456015.3.0000.5274). All patients provided written consent. Patients were eligible for inclusion if they were histologically diagnosed with ER‐positive breast cancer, received 20 mg tamoxifen daily for at least 3 months, and were not concomitantly taking moderate or strong inhibitors or inducers of CYP2D6, CYP3A, and the drug transporter ABCB1 (P‐glycoprotein). Blood samples were collected at steady‐state, immediately before the administration of the next scheduled daily dose of tamoxifen, to ensure measurement of the steady‐state plasma concentration (C_p_, ss). Blood samples were collected in tubes containing heparin within a 24‐h interval. Plasma samples were kept at −80°C until analysis.

### Plasma Sample Preparation and Measurement

Plasma samples of 200 µL were spiked with 25 µL of internal standard (IS) solution (4 µg/mL of mexiletine), 25 µL of 1 M sodium hydroxide, and 2 mL of tert‐butyl methyl ether and were mixed on a shaker table (300 ± 10 cycles/min) for 30 min. Samples were then centrifuged for 10 min at 1800 × g to separate the aqueous phase from the organic one; the latter was then removed and dried at room temperature under an airstream. The dry residue was then dissolved in 100 µL of the mobile phase.

Tamoxifen, endoxifen, 4‐hydroxy‐tamoxifen, N‐desmethyltamoxifen, and mexiletine (IS) were resolved on an RP‐Select B LiChroCART column using ammonium formate 10 mM and acetonitrile, both acidified with 0.1% formic acid, as mobile phase in a 1:1 (v/v) proportion. The compounds were quantified using a tandem mass spectrometry system consisting of a Quattro Micro LC triple‐quadrupole (Waters, Milford, Massachusetts, USA) equipped with an electrospray ionization (ESI) interface and operated in positive ion mode. The method was validated according to the FDA Bioanalytical Method Validation Guidance for Industry and EMA Bioanalytical Method Validation^17^, ^18^ showing coefficients of variation and the relative standard errors of the accuracy and precision studies less than 15%, a linearity of 1‐1250 ng/mL for endoxifen and tamoxifen, 2‐2500 ng/mL for N‐desmethyltamoxifen, and 0.4‐500 ng/mL for 4‐hydroxy‐tamoxifen (Supplemental Information S1).

### CYP2D6 Genotyping and Inference of Phenotyping

DNA was obtained from peripheral blood according to usual procedures with a QIAamp DNA Blood Mini Kit (Qiagen, Hilden, GER) according to the manual's instructions. For the *CYP2D6*, the following SNPs were detected by real‐time polymerase chain reaction with 5‐nuclease allelic discrimination assays according to the manufacturer's instructions: 1584C > G (rs1080985), 31G > A (rs769258), 100C > T (rs1065852), 1023C > T (rs28371706), 1846G > A (rs3892097), 2549A > del (rs35742686), 2615_ 2617delAAG (rs28371720), 2850C > T (rs16947), 2988G > A (rs28371725), 3183G > A (rs59421388), 4180G > C (rs1135840). Gene deletion (CYP2D6*5, Hs00010001_cn) and duplication/multiplication (CYP2D6*xN) were analyzed by TaqMan copy number assay.

As described previously by the present group,^19^ CYP2D6 diplotypes were inferred using HaploStats software (version 1.7.7) from software R. For the star allele (*) designation, the software‐generated haplotypes were compared to the PharmVar Database (https://www.pharmvar.org/gene/CYP2D6). The CYP2D6*1 allele was attributed when no nucleotide change was observed in all genotyped SNPs. The phenotype was determined using the activity score (AS) system, according to the Final Consensus for CYP2D6 genotype to phenotype assignment, adopted by the Clinical Pharmacogenetics Implementation Consortium (https://cpicpgx.org/resources/cyp2d6‐genotype‐to‐phenotype‐standardization‐project/).

### Data and Statistical Analysis

The geometric mean and corresponding 95% confidence intervals of the steady‐state plasma concentrations for tamoxifen and its metabolites were calculated using R software (version 4.4.0). The normality of the distribution of the steady‐state plasma (C_p,ss_) concentration for each compound was assessed using normal probability plots and the Shapiro–Wilk test implemented in the stats package. The sample size was estimated using endoxifen plasma concentration data reported previously^20^ (C_max_ − mean ± standard deviation: 5.56 ± 2.14 ng/mL). Calculations were performed with the online tool *PS: Power and Sample Size Calculation* (https://cqsclinical.app.vumc.org/ps). Based on these data, enrolling 13 patients per group would provide 80% power (β =.20) to detect a 50% difference in endoxifen plasma concentration between CYP2D6 normal metabolizers (NM) and intermediate metabolizer (IM), at a two‐sided significant level of 5% (α = 0.05), we aimed to recruit more than 26 participants to ensure sufficient representation.

Comparisons of tamoxifen and its metabolites steady‐state plasma concentrations between phenotype groups (IM and NMs) were performed using the Wilcoxon test. A two‐way ANOVA was conducted to evaluate potential interactions between phenotype and age groups on steady‐state plasma concentration (C_p,ss_) of tamoxifen and metabolites. Additionally, the Kruskal–Wallis test was employed to compare endoxifen steady‐state plasma concentrations (C_p,ss_) among NMs stratified by activity scores (1.25, 1.5, and 2). For all analyses, the significance level of.05 was applied.

## Results

**Figure 1:**
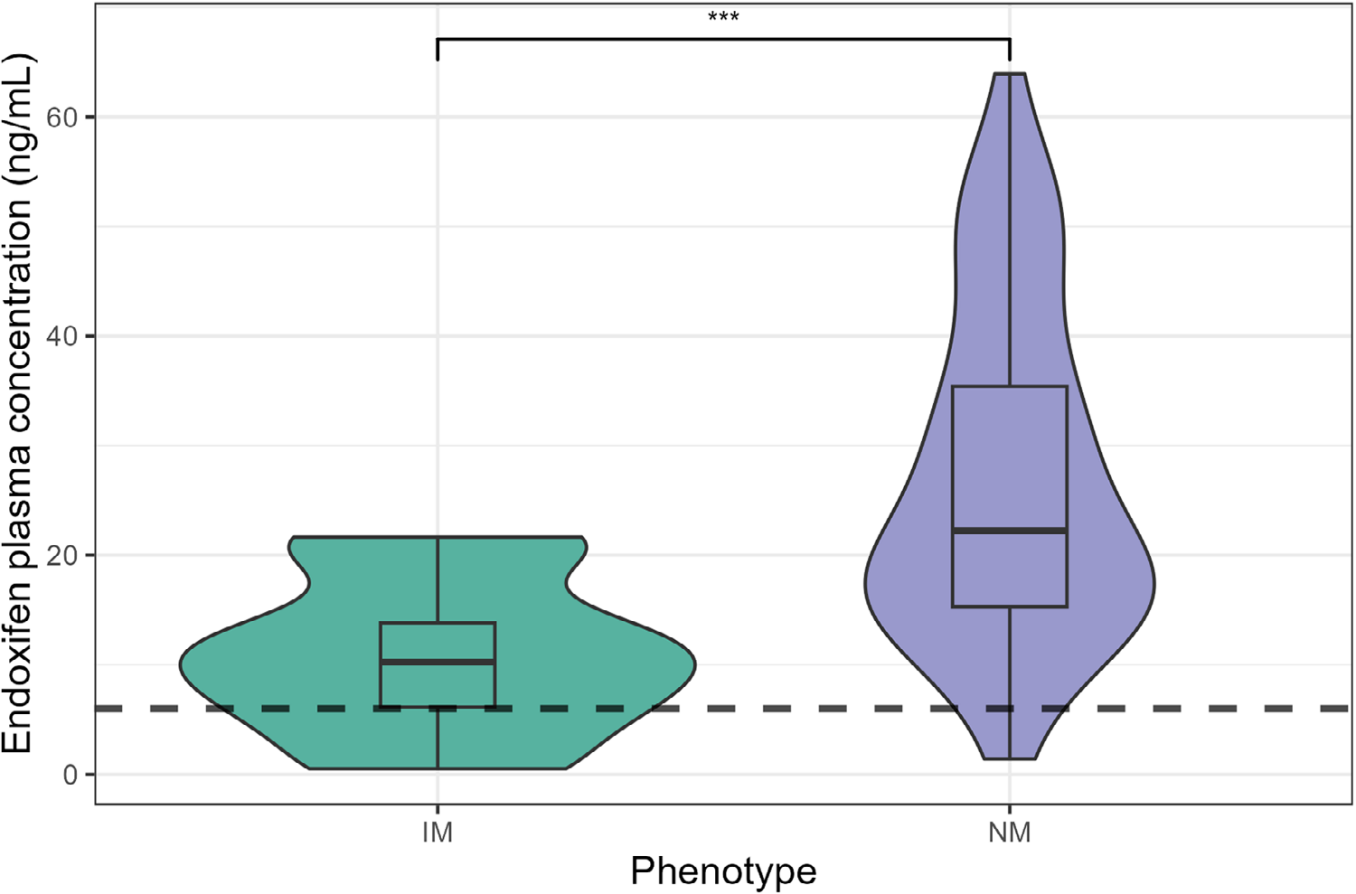
Violin plot showing the distribution of endoxifen steady‐state plasma concentrations stratified by CYP2D6 activity score‐derived metabolizer phenotype (IM, intermediate metabolizers; NM, normal metabolizers). The dashed line represents the proposed efficacy threshold of 6 ng/mL (n = 63).

The study cohort consisted of 63 breast cancer patients after provided written consent. None of the participants were using moderate or strong inhibitors or inducers of CYP2D6, CYP3A, or the drug transporter P‐glycoprotein. Patient ages ranged from 34 to 79 years, with a mean age of 51.1 years; 55 patients were older than 40 years, and 8 were 40 years old or younger. CYP2D6 genotyping classified 13 patients as IMs, 49 as NMs, and one as an ultrarapid metabolizer (UM), based on the calculated activity score. The steady‐state plasma concentration (Cp, ss) of endoxifen was significantly lower in IMs compared to NMs, as shown in Table 1. The geometric mean concentration of endoxifen in the IM group was 7.13  ng/mL (95% CI: 3.38‐15.08), whereas in the NM group, it was 22.66  ng/mL (95% CI: 18.57‐27.66), corresponding to a 2.18‐fold higher concentration in NMs (*P*  <.001). Furthermore, 23.1% of IM patients exhibited endoxifen levels below the therapeutic threshold of 6  ng/mL, in contrast to only 4.1% of NM patients (Figure 1).

**Table 1:** Geometric Mean (95% CI) of Steady‐State Plasma Concentrations of Tamoxifen and Its Metabolites in Breast Cancer Patients, Stratified According to CYP2D6 Activity Score‐Derived Metabolizer Phenotype

	Total n = 63	UM n = 1	NM n = 49	IM n = 13	*P* Value
Activity score		>2.25	1.25‐2.25	0.5‐1.0	
C_p, SS_ (ng/mL)					
Tamoxifen	142.09 (125.67‐160.64)	113.36	150.61 (133.31‐170.15)	116.08 (77.62‐173.58)	.215
Endoxifen	18.00 (14.17‐22.87)	38.59	22.66 (18.57‐27.66)	7.13 (3.07‐15.08)	<.001
4‐Hydroxy‐tamoxifen	3.86 (3.37‐4.42)	3.53	4.15 (0.13‐3.63)	2.96 (1.89‐4.62)	.116
N‐Desmethyltamoxifen	718.77 (622.94‐829.34)	617.29	775.86 (677.12 ‐ 889.00)	545.19 (333.58‐891.03)	.166

C_p, SS_, steady‐state plasma concentrations; IM, intermediate metabolizer; NM, normal metabolizer; UM, ultrarapid metabolizer. *P* value from Wilcoxon test between IM and NM groups.

**Figure 2:**
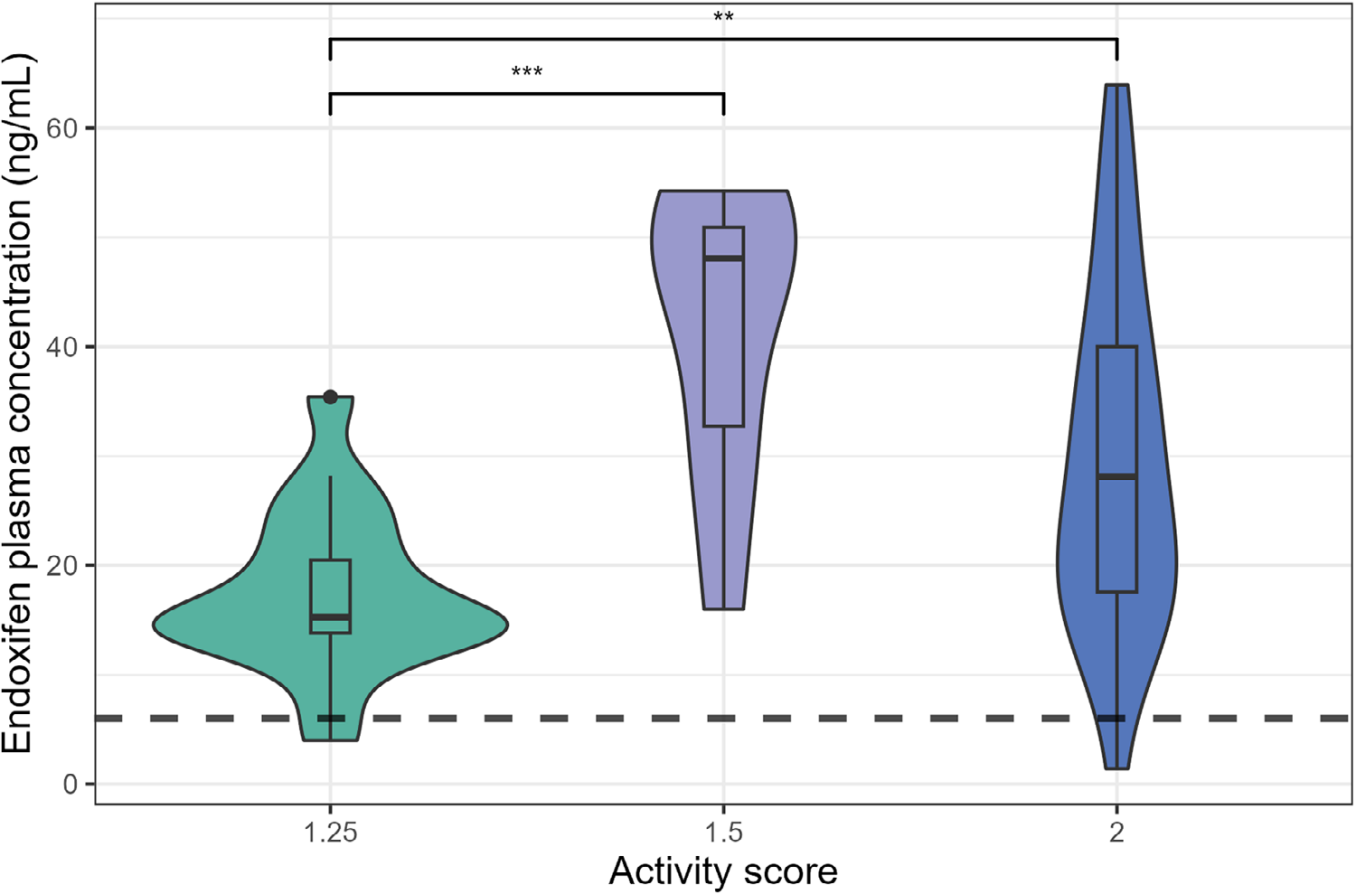
Violin plot showing the distribution of endoxifen steady‐stage plasma concentrations stratified by the activity score range of the normal metabolizer phenotype. The dashed line indicates the proposed efficacy threshold of 6 ng/mL (n = 49).

The geometric mean steady‐state plasma concentrations (Cp, ss) of endoxifen among NMs with activity scores of 1.25, 1.5, and 2 were 15.86 ng/mL (95% CI: 12.29‐20.47), 38.80 ng/mL (95% CI: 27.29‐55.17), and 24.38 ng/mL (95% CI: 17.68‐33.63), respectively. When comparing these geometric means, a significant difference was observed between activity scores 1.25 and 1.5 (*P* <.01), as well as between scores 1.25 and 2 (*P* <.01; Figure 2). The proportion of patients with endoxifen levels below the therapeutic threshold of 6  ng/mL was 5.88% among those with an activity score of 1.25 (n = 17) and 4.17% among those with an activity score of 2 (n = 24); no patients with an activity score of 1.5 (n = 8) exhibited concentrations below 6  ng/mL (Figure 2).

Regarding the other metabolites, no statistically significant differences were observed between NM and IM groups (Table 1). The geometric mean steady‐state plasma concentrations (C_p_, SS) of tamoxifen, 4‐hydroxy‐tamoxifen, and N‐desmethyltamoxifen were 150.61, 4.15, and 775.86  ng/mL, respectively, in NM patients, and 116.08, 2.96, and 545.19  ng/mL, respectively, in IM patients (*P* values:.215,.116, and.166, respectively). Metabolic ratios between tamoxifen and each of its metabolites are presented in Table 2. Among these, only the tamoxifen/endoxifen ratio showed a statistically significant difference, with a higher value in the IM group compared to the NM group (16.27 versus 6.65; *P* = .048).

**Table 2:** Metabolic Ratios of Tamoxifen to Its Metabolites in Breast Cancer Patients, Stratified by CYP2D6 Activity Score Phenotype

Metabolic Ratios	Total n = 63	UM n = 1	NM n = 49	IM n = 13	*P* Value
Tamoxifen/endoxifen	7.89 (6.48‐9.62)	2.94	6.65 (5.48‐8.06)	16.27 (10.18‐26.02)	.048
Tamoxifen/4‐hydroxy‐tamoxifen	36.83 (33.37‐40.64)	32.11	36.31 (32.47‐40.59)	39.26 (30.39‐50.71)	.564
Tamoxifen/N‐desmethyltamoxifen	0.19 (0.18‐0.22)	0.18	0.19 (0.17‐0.22)	0.21 (0.16‐0.28)	.745

NM, normal metabolizer; UM, ultrarapid metabolizer.

*P* value from Wilcoxon and t‐test between IM and NM groups.

When stratified by age, 7.3% of patients older than 40 years and 12.5% of those aged 40 years or younger exhibited endoxifen steady‐state concentrations below the efficacy threshold of 6 ng/mL. Despite this difference in the proportion of patients with subtherapeutic levels, the sample size was insufficient to detect a statistically significant difference in the geometric mean steady‐state plasma concentrations of endoxifen between the two age groups (18.06  ng/mL for patients > 40 years versus 17.65  ng/mL for those ≤ 40 years).

## Discussion

In the present study, steady‐state plasma concentrations (Cp, ss) of tamoxifen and its metabolites (endoxifen, 4‐hydroxy‐tamoxifen, and N‐desmethyltamoxifen) were evaluated in 63 Brazilian female breast cancer patients. CYP2D6 phenotypes were determined based on individual activity scores. The findings suggest that combining CYP2D6 phenotyping with TDM of endoxifen may represent an effective strategy to guide tamoxifen dose optimization and ensure adequate drug exposure in this population.

The metabolism and activation of tamoxifen involve several enzymes, including CYP2C19, CYP2C9, CYP2B6, and CYP1A2, but are primarily mediated by CYP3A4/5 and CYP2D6. Although different studies have evaluated the influence of other enzymes on endoxifen formation and its high interindividual variability, CYP2D6 remains the main enzyme responsible for endoxifen production, with only a minor contribution from CYP3A4/5.^21^, ^22^ In the overall cohort of this study, the geometric mean steady‐state plasma concentrations of tamoxifen, endoxifen, 4‐hydroxy‐tamoxifen, and N‐desmethyltamoxifen (Table 1) were consistent with values reported in previous studies conducted in breast cancer patients.^5^, ^16^, ^23^

The metabolic ratios presented in Table 2 are slightly different from those reported previously^24^ with 75 breast cancer patients undergoing tamoxifen treatment, with values of 5.7 for tamoxifen/endoxifen, 22.5 for tamoxifen/4‐hydroxy‐tamoxifen, and 0.5 for tamoxifen/N‐desmethyltamoxifen. In another study^25^ conducted with 10 breast cancer patients, the metabolic ratios were 11.9 for tamoxifen/endoxifen, 71 for tamoxifen/4‐hydroxy‐tamoxifen, and 0.5 for tamoxifen/N‐desmethyltamoxifen.

The range found in the present study (63 patients) from lowest to highest plasma concentration at steady‐state for tamoxifen (24.69 and 326.09 ng/mL) and endoxifen (0.51 and 63.93 ng/mL) following the tamoxifen fixed dose of 20 mg/day demonstrated significant interindividual variability among the patients who receive this adjuvant breast cancer treatment, as previously related by other authors^9^, ^10^, ^24^ and which can influence in the treatment outcome.

In a previous study, the most frequent CYP2D6 phenotype in the Brazilian population was NMs at 83.5%, followed by IMs at 10.3%, poor metabolizer (PM) at 3.7%, and UM at 2.5%.^26^ In the present study, the distribution of CYP2D6 phenotypes was similar, with 77.78% classified as NM, 20.63% as IM, and 1.59% as UM, corresponding to one patient among the 63 evaluated. For this UM patient, the steady‐state plasma concentration of endoxifen was 38.59 ng/mL, which was above the proposed efficacy threshold and approximately 0.70‐fold higher than the mean concentration observed in NM patients, a finding consistent with a previous study.^27^

Plasma concentrations below the efficacy threshold of 6 ng/mL^9^ were 4.08% among NM patients and 23.08% for IM patients. Furthermore, the difference between NM and IM groups was statistically significant, which can be explained by the fact that the IM CYP2D6 phenotype is related to a lower CYP2D6 activity.^12^ Similar results were obtained previously^16^, ^28^, ^29^, ^30^ where patients with the IM or PM phenotype for the CYP2D6 presented lower concentrations for endoxifen when compared to NM or UM groups.

In a previous study, a substantial proportion of patients exhibited endoxifen plasma concentrations below the efficacy threshold of 6 ng/mL during follow‐up after initiating tamoxifen therapy.^16^ To address subtherapeutic levels, some patients underwent dose escalation, while others were switched to an aromatase inhibitor. Notably, 100% of PMs and 36% of IMs had concentrations below the threshold. In the present study, the proportion of patients with subtherapeutic endoxifen levels was low across all phenotype groups. Additionally, differences in geometric mean endoxifen concentrations were observed among NMs when stratified by activity score.

Therefore, the current study supports the TDM for breast cancer patients under tamoxifen treatment, since the effectiveness of the therapy is related to the levels of endoxifen, which is dependent on several unknown factors that go beyond CYP2D6 phenotype.^30^, ^31^ Furthermore, according to a previous study of our research group,^32^ patients can be monitored at any time of dose interval after three months of treatment, whereas endoxifen showed similar plasma concentrations in all serial samples collected during a 24‐h dose interval.

This study presents certain limitations. First, none of the patients included in the present cohort were phenotyped as CYP2D6 PMs, which may limit the generalizability of the findings to this subgroup. Second, the sample size (n = 63) might be considered relatively small for certain statistical analyses, particularly for the age subgroup analysis, in which only eight patients were younger than 40 years. Third, although the validated analytical method was developed to quantify the three major metabolites of tamoxifen, more than 36 metabolites have been described to date,^30^ potentially restricting a comprehensive metabolic profile. Fourth, genetic ancestry data were not collected at the time of recruitment, preventing the performance a principal component analysis, limiting the assessment of population stratification and genotype frequencies according to ancestry. Finally, the plasma concentration measurements reflected the combined levels of the isomeric mixtures of (E)‐ and (Z)‐endoxifen, as well as (E)‐ and (Z)‐4‐hydroxy‐tamoxifen, without distinguishing individual isomers.

## Conclusions

In conclusion, substantial interindividual variability in endoxifen plasma concentrations was observed among breast cancer patients after 3 months of tamoxifen treatment (20 mg daily). Although CYP2D6 genotyping plays a critical role at the initiation of therapy, 4.08% of NMs and 23.08% of IMs presented endoxifen plasma concentrations below the proposed efficacy threshold. These findings suggest that a combined approach integrating CYP2D6 genotyping and TDM may represent the most effective strategy to identify patients at risk of subtherapeutic exposure and to guide personalized tamoxifen therapy.

## Conflicts of Interest

All authors declared no competing interest for this work.

## Funding

The authors thank the participants of this study. This study was funded by the São Paulo Research Foundation (FAPESP, 2014/16360‐9), the Conselho Nacional de Desenvolvimento Científico e Tecnológico (CNPq), and the Coordenação de Aperfeiçoamento de Pessoal em Nível Superior (CAPES).

## Supporting information



Supporting Information

## Data Availability

The data supporting the findings of this study are available from the corresponding author upon reasonable request.

## References

[1] Bray F , Laversanne M , Sung H , et al. Global cancer statistics 2022: GLOBOCAN estimates of incidence and mortality worldwide for 36 cancers in 185 countries. CA Cancer J Clin. 2024;74(3):229‐263. doi:10.3322/caac.21834 38572751

[2] Fohlin H , Nordenskjöld A , Rosell J , et al. Breast cancer hormone receptor levels and benefit from adjuvant tamoxifen in a randomized trial with long‐term follow‐up. Acta Oncol. 2024;63:535‐541. doi:10.2340/1651-226X.2024.40493 38967128 PMC11332493

[3] (EBCTCG) EBCTCG , Davies C , Godwin J , et al. Relevance of breast cancer hormone receptors and other factors to the efficacy of adjuvant tamoxifen: patient‐level meta‐analysis of randomised trials. Lancet. 2011;378(9793):771‐784. doi:10.1016/S0140-6736(11)60993-8 21802721 PMC3163848

[4] Howell A , Howell SJ . Tamoxifen evolution. Br J Cancer. 2023;128(3):421‐425. doi:10.1038/s41416-023-02158-5 36765172 PMC9938251

[5] de Vries Schultink AHM , Huitema ADR , Beijnen JH . Therapeutic Drug Monitoring of endoxifen as an alternative for CYP2D6 genotyping in individualizing tamoxifen therapy. The Breast. 2018;42:38‐40. doi:10.1016/j.breast.2018.08.100 30153552

[6] Skaar TC , Desta Z . CYP2D6 and endoxifen in tamoxifen therapy: a tribute to David A. Flockhart. Clin Pharmacol Ther. 2018;103(5):755‐757. doi:10.1002/cpt.1039 29473149

[7] Lim JSL , Sutiman N , Muerdter TE , et al. Association of CYP2C19*2 and associated haplotypes with lower norendoxifen concentrations in tamoxifen‐treated Asian breast cancer patients. Br J Clin Pharmacol. 2016;81(6):1142‐1152. doi:10.1111/bcp.12886 26799162 PMC4876188

[8] Jordan VC , Collins MM , Rowsby L , Prestwich G . A monohydroxylated metabolite of tamoxifen with potent antioestrogenic activity. J Endocrinol. 1977;75(2):305‐316. doi:10.1677/joe.0.0750305 591813

[9] Madlensky L , Natarajan L , Tchu S , et al. Tamoxifen metabolite concentrations, CYP2D6 genotype, and breast cancer outcomes. Clin Pharmacol Ther. 2011;89(5):718‐725. doi:10.1038/clpt.2011.32 21430657 PMC3081375

[10] Mürdter TE , Schroth W , Bacchus‐Gerybadze L , et al. Activity levels of tamoxifen metabolites at the estrogen receptor and the impact of genetic polymorphisms of phase I and II enzymes on their concentration levels in plasma. Clin Pharmacol Ther. 2011;89(5):708‐717. doi:10.1038/clpt.2011.27 21451508

[11] Braal CL , Westenberg JD , Buijs SM , et al. Factors affecting inter‐individual variability in endoxifen concentrations in patients with breast cancer: results from the prospective TOTAM trial. Breast Cancer Res Treat. 2022;195(1):65‐74. doi:10.1007/s10549-022-06643-y 35842520 PMC9338137

[12] Goetz MP , Sangkuhl K , Guchelaar HJ , et al. Clinical Pharmacogenetics Implementation Consortium (CPIC) Guideline for CYP2D6 and tamoxifen therapy. Clin Pharmacol Ther. 2018;103(5):770‐777. doi:10.1002/cpt.1007 29385237 PMC5931215

[13] Fox P , Balleine RL , Lee C , et al. Dose escalation of tamoxifen in patients with low endoxifen level: evidence for therapeutic drug monitoring—the TADE Study. Clin Cancer Res. 2016;22(13):3164‐3171. doi:10.1158/1078-0432.ccr-15-1470 26847054

[14] Binkhorst L , Mathijssen RHJ , Jager A , van Gelder T . Individualization of tamoxifen therapy: much more than just CYP2D6 genotyping. Cancer Treat Rev. 2015;41(3):289‐299. doi:10.1016/j.ctrv.2015.01.002 25618289

[15] Saladores P , Mürdter T , Eccles D , et al. Tamoxifen metabolism predicts drug concentrations and outcome in premenopausal patients with early breast cancer. Pharmacogenomics J. 2015;15(1):84‐94. doi:10.1038/tpj.2014.34 25091503 PMC4308646

[16] Braal CL , Jager A , de Hoop EO , et al. Therapeutic drug monitoring of endoxifen for tamoxifen precision dosing: feasible in patients with hormone‐sensitive breast cancer. Clin Pharmacokinet. 2022;61(4):527‐537. doi:10.1007/s40262-021-01077-z 34786650 PMC8975771

[17] Food and Drug Administration (FDA) . Bioanalytical Method Validation: Guidance for Industry. 2018. https://www.fda.gov/files/drugs/published/Bioanalytical‐Method‐ValidationGuidance‐for‐Industry.pdf

[18] European Medicines Agency (EMA) . Guideline on Bioanalytical Method Validation. 2011. https://www.ema.europa.eu/en/documents/scientific‐guideline/guideline‐bioanalyticalmethod‐validation_en.pdf 10.4155/bio.12.44 22533559

[19] Ximenez JPB , De Andrade JM , Marques MP , Coelho EB , Suarez‐Kurtz G , Lanchote VL . Hormonal status affects plasma exposure of tamoxifen and its main metabolites in tamoxifen‐treated breast cancer patients. BMC Pharmacol Toxicol. 2019;20(Suppl 1):81. doi:10.1186/s40360-019-0358-y 31852530 PMC6921430

[20] Kanji CR , Nyabadza G , Nhachi C , Masimirembwa C . Pharmacokinetics of tamoxifen and its major metabolites and the effect of the African ancestry specific CYP2D6*17 variant on the formation of the active metabolite, endoxifen. J Pers Med. 2023;13(2):272. doi:10.3390/jpm13020272 36836506 PMC9961245

[21] Sanchez‐Spitman AB , Swen JJ , Dezentjé VO , Moes D , Gelderblom H , Guchelaar HJ . Effect of CYP2C19 genotypes on tamoxifen metabolism and early‐breast cancer relapse. Sci Rep. 2021;11(1):415. doi:10.1038/s41598-020-79972-x 33432065 PMC7801676

[22] Sanchez Spitman AB , Moes D , Gelderblom H , Dezentje VO , Swen JJ , Guchelaar HJ . Effect of CYP3A4*22, CYP3A5*3, and CYP3A combined genotypes on tamoxifen metabolism. Eur J Clin Pharmacol. 2017;73(12):1589‐1598. doi:10.1007/s00228-017-2323-2 28849250 PMC5684327

[23] EL Desoky ES , Taha AF , Mousa HS , et al. Value of therapeutic drug monitoring of endoxifen in Egyptian premenopausal patients with breast cancer given tamoxifen adjuvant therapy: a pilot study. J Oncol Pharm Pract. 2022;29(7):1673‐1686. doi:10.1177/10781552221146531 36567618

[24] Jager NGL , Rosing H , Linn SC , Schellens JHM , Beijnen JH . Importance of highly selective LC‐MS/MS analysis for the accurate quantification of tamoxifen and its metabolites: focus on endoxifen and 4‐hydroxytamoxifen. Breast Cancer Res Treat. 2012;133(2):793‐798. doi:10.1007/s10549-012-2000-1 22388692 PMC3362711

[25] Teunissen SF , Jager NGL , Rosing H , Schinkel AH , Schellens JHM , Beijnen JH . Development and validation of a quantitative assay for the determination of tamoxifen and its five main phase I metabolites in human serum using liquid chromatography coupled with tandem mass spectrometry. J Chromatogr B. 2011;879(19):1677‐1685. doi:10.1016/j.jchromb.2011.04.011 21543272

[26] Friedrich DC , Genro JP , Sortica VA , et al. Distribution of CYP2D6 alleles and phenotypes in the Brazilian population. PLoS One. 2014;9(10):e110691. doi:10.1371/journal.pone.0110691 25329392 PMC4203818

[27] Puszkiel A , Arellano C , Vachoux C , et al. Model‐based quantification of impact of genetic polymorphisms and co‐medications on pharmacokinetics of tamoxifen and six metabolites in breast cancer. Clin Pharmacol Ther. 2021;109(5):1244‐1255. doi:10.1002/cpt.2077 33047329

[28] Souwer ETD , Sanchez‐Spitman A , Moes D , et al. Tamoxifen pharmacokinetics and pharmacodynamics in older patients with non‐metastatic breast cancer. Breast Cancer Res Treat. 2023;199(3):471‐478. doi:10.1007/s10549-023-06925-z 37067610 PMC10175413

[29] Medwid S , Schwarz UI , Choi Y , Keller D , Ross C , Kim RB . Solanidine metabolites as diet‐derived biomarkers of CYP2D6‐mediated tamoxifen metabolism in breast cancer patients. Clin Pharmacol Ther. 2024;116(5):1269‐1277. Published online 2024. doi:10.1002/cpt.3380 39039708

[30] Antunes MV , Linden R , Santos TV , et al. Endoxifen levels and its association with CYP2D6 genotype and phenotype: evaluation of a Southern Brazilian population under tamoxifen pharmacotherapy. Ther Drug Monit. 2012;34(4):422‐431. 22777153 10.1097/FTD.0b013e318260b46e

[31] Sanchez‐Spitman AB , Böhringer S , Dezentjé VO , Gelderblom H , Swen JJ , Guchelaar H . A genome‐wide association study of endoxifen serum concentrations and adjuvant tamoxifen efficacy in early‐stage breast cancer patients. Clin Pharmacol Ther. 2024;116(1):155‐164. doi:10.1002/cpt.3255 38501904

[32] Ximenez JP , Lanchote VL , Bello MA , et al. Post‐marketing assessment of generic tamoxifen in Brazilian breast cancer patients. Basic Clin Pharmacol Toxicol. 2019;126(5):432‐436. doi:10.1111/bcpt.13368 31758654

