# Supplementary material for: From Genotype to Therapeutic Monitoring: Enhancing Tamoxifen Efficacy in Breast Cancer Treatment
Source: J Clin Pharmacol. 2025 Sep 5;65(12):1712–8. doi: 10.1002/jcph.70103 (PMC12649294; doi:10.1002/jcph.70103)
Supplement: Supplementary file 1 — Supporting Information [file JCPH-65-1712-s001.pdf]

Supplementary Files

From Genotype to Therapeutic Monitoring: Enhancing Tamoxifen Efficacy in Breast Cancer Treatment

Ana Flávia Mendes Batista (1), Letícia Penteado Petrolli (1); Maria Paula Marques Pereira, PhD (1); Adriana Rocha, PhD (1); Jurandyr Moreira de Andrade, PhD (2); Vera Lucia Lanchote, PhD (1); João Paulo Ximenez, PhD (1)

(1) School of Pharmaceutical Sciences of Ribeirao Preto, University of Sao Paulo, Ribeirao Preto, Brazil
(2) School of Medicine of Ribeirao Preto, University of Sao Paulo, Ribeirao Preto, Brazil

1. Plasma sample preparation and measurement

1.1. Plasma sample preparation

Two hundred microliters of plasma samples spiked with 25 µL of IS solution (4 µg/mL of mexiletine), 25 µL of 1 M sodium hydroxide, and 2 mL of tert-Butyl methyl ether were mixed on a shaker table (300±10 cycles/min) for 30 min. Samples were then centrifuged for 10 min at 1800 × g to separate the aqueous phase from the organic one, the latter was then removed and dried at room temperature under an airstream. The dry residue was then dissolved in 100 µL of the mobile phase.

2. LC-MS/MS

2.1. Chromatographic conditions

An RP-Select B LiChroCART® column 5 µm, 125 mm × 4 mm, (LiChrospher®, Merck, Darmstadt, GER) was used to separate tamoxifen and its metabolites endoxifen, N-desmethyltamoxifen and 4-hydroxy-tamoxifen maintained at a temperature of 24 °C. Aqueous ammonium formate 10 mM (purity: 99%, Sigma-Aldrich, St Louis, USA) and acetonitrile (purity: 99,9%, Merck, Darmstadt, GER), both acidified with 0.1% formic acid (purity: 88%, JT Baker, Mexico City, Mexico), were used as mobile in 1:1 (v/v) proportion. An autosampler

(at 12°C) injected volumes of 60 μL onto the LC column. The overall run time was 20 min. The column effluent was introduced to the mass spectrometer and monitored.

2.2. MS detection

The tandem mass spectrometry system consisted of a Quattro Micro API® (Waters, Milford, Massachusetts, USA) equipped with an electrospray ionization (ESI) interface and operated in positive ion mode. The mass spectrometer was used in the multiple-reaction monitoring (MRM) mode, the protonated tamoxifen, endoxifen, 4-hydroxy-tamoxifen, N-desmethyltamoxifen and internal standard mexiletine $[M+H]^+$ and their respective product ions were monitored at transitions of 372,6 > 71,9 *m/z*; 374,9 > 57,8 *m/z*; 388,7 > 71,9 *m/z*; 358,9 > 57,5 *m/z* and 180 > 58 *m/z*, respectively. The temperature of the ESI source during the run was respectively set at 120°C (for the source) and 250°C (for the desolvation gas), and the ion spray voltage was kept at 3 kV. The desolvation gas was set at 1000 L/h, the cone gas at 150 L/h (nitrogen), the collision gas (argon) at 0.19 mL/min. The MassLynx V4.1 software (Waters, Milford, Massachusetts, USA) was used for control of the MS system, the acquisition and processing of data. Quantification was performed using QuanLynx as implemented in the MassLynx software.

3. Method validation

The LC–MS/MS method was validated according to the FDA Bioanalytical Method Validation Guidance for Industry [1] and EMA Bioanalytical Method Validation [2].

Calibration samples and quality control points at LLOQ, LQC, MQC and HQC concentrations, and DQC were prepared in blank plasma from the stock solutions and used for determination of linearity, precision and accuracy of the method.

Calibration curves were constructed by correlating peak area ratio for each compound (versus mexiletine used as internal standard) as a function of the concentration of the spiked

standard solutions. Calibration points were in the interval from 1 to 1250 ng/mL for endoxifen and tamoxifen; from 2 to 2500 ng/mL for NDTAM and from 0.4 to 500 ng/mL for 4-hydroxy-tamoxifen. They were prepared in triplicate, using 200 µL blank plasma enriched with 25 µL of standard solutions of each compound, including blank sample and zero sample.

Selectivity was determined using 200 µL blank plasma obtained from 8 different healthy participants (4 normal, 2 lipemic and 2 hemolyzed). The chromatograms obtained were compared to the LLOQ samples. For the carryover effect, blank plasma injections were analyzed before and after injecting the upper limit of quantification (ULOQ) sample. The chromatogram obtained after injection of ULOQ sample was compared with LLOQ sample chromatogram.

Matrix effect was evaluated using 200 µL blank plasma obtained from 8 different healthy participants (4 normal, 2 lipemic and 2 hemolyzed). The blank plasma extracts were enriched with IS and standard solutions corresponding to 2,5 and 1250 ng/mL of tamoxifen and endoxifen; 0,4 and 500 ng/mL of 4-hydroxy-tamoxifen; 5 and 2500 ng/mL of NDTAM. The same standard solutions were prepared in methanol and added IS. For each sample was calculated the internal standard normalized matrix factor (NMF) comparing the analytes in plasma with those in methanol at the same concentrations, according to the equation below.

$$NMF = \frac{area\ of\ matrix\ /area\ of IS\ in\ matrix}{area\ of\ analyte\ in\ solution\ /area\ of IS\ in\ solution}$$

The efficiency of the extraction process was determined using 8 different sources of blank plasma enriched with the same concentration of standard solutions of the matrix effect. The results were compared with those of standard IS solutions added with methanol and the percentage of recovery was evaluated based on the normalized results, using the following equation:

$$Recovery = \left(\frac{area\ of\ extracted\ analyte\ /\ area\ of\ extracted\ IS}{area\ of\ analyte\ in\ methanol\ /\ area\ of\ IS\ in\ methanol}\right) * 100$$

Precision and accuracy were determined using 6 replicates of LLOQ, LQC, HQC, and DQC analyzed in a single analytical run (intra-assay) and in 3 separate runs (inter-assay). Results were expressed as coefficient of variation (CV) and relative standard error (RSE).

$$RSE = \left(\frac{mean\ experimental\ concentration\ -\ nominal\ concentration}{nominal\ concentration}\right) * 100$$

For the evaluation of stability in plasma samples after multiple freeze-thaw cycles, LQC and HQC underwent three freeze/thaw cycles. Frozen samples were allowed to thaw at room temperature for 2h and were subsequently refrozen at −80°C during approximately 24h. Tamoxifen and its metabolites levels were measured in aliquots from the three-consecutive freeze–thaw cycles.

Table S1 - Quality controls concentrations of tamoxifen and its metabolites.

Sample (ng/mL)	tamoxifen	endoxifen	4-hydroxy-tamoxifen	N-desmethyltamoxifen
**LLOQC**	1	1	0.4	2
**LQC**	3	3	1.2	6
**MQC**	400	400	160	800
**HQC**	1000	1000	400	2000
**DQC**	625	625	250	1250

LLOQC, LQC, MQC, HQC, and DQC are the quality controls of the lower limit of quantification, low, medium, high concentration, and dilution.

Table S2. Confidence limits of the analytical method of TAM and its metabolites endoxifen, 4-hydroxy-tamoxifen and N-desmethyltamoxifen in human plasma samples.

	tamoxifen	endoxifen	4-hydroxy-tamoxifen	N-desmethyltamoxifen
**Matrix Effect (CV%)**	10.78	6.14	8.25	8.24
**Quantification limit**				
LLOQC (n = 10)	1 ng/mL	1 ng/mL	0.4 ng/mL	2 ng/mL
Precision (CV%)	9.58	18.64	19.40	20.12
Accuracy (RSE%)	0.60	1.40	0.75	3.25
**Linearity**	1 – 1250 ng/mL	1 – 1250 ng/mL	0.4 – 500 ng/mL	2 – 2500 ng/mL
r	0.9952	0.9907	0.9969	0.9951
Equation	0.0291x + 0.0152	0.0123x + 0.0143	0.0363x + 0.0038	0.0052x + 0.0110
**Precision Intra-assay (CV%)**				
LLOQC (n = 5)	7.18	4.50	6.07	12.02
LQC (n = 5)	3.43	9.60	6.90	3.59
MQC (n = 5)	7.88	1.37	8.35	7.33
HQC (n = 5)	3.01	8.05	1.72	2.52
DQC (n = 5)	4.26	5.35	2.30	3.16
**Precision Inter-assay (CV%)**				
LLOQC (n = 15)	9.84	9.07	12.31	8.05
LQC (n = 15)	4.67	8.45	8.13	8.43
MQC (n = 15)	6.26	4.86	7.79	7.73
HQC (n = 15)	9.56	5.39	5.12	9.02
DQC (n = 15)	4.12	6.93	4.59	5.95
**Accuracy Intra-assay (RSE%)**				
LLOQC (n = 5)	7.00	-2.40	10.50	-7.80
LQC (n = 5)	-1.47	-0.47	2.00	-7.33
MQC (n = 5)	-0.94	1.09	12.60	7.33
HQC (n = 5)	-1.59	-6.45	2.40	4.76
DQC (n = 5)	-2.63	-9.20	-1.49	1.76
**Accuracy Inter-assay (RSE%)**				
LLOQC (n = 15)	-2.47	-1.00	-3.33	-7.30
LQC (n = 15)	0.76	-0.42	1.94	-0.07
MQC (n = 15)	1.44	-1.39	7.77	2.05
HQC (n = 15)	1.30	-5.73	4.03	1.45
DQC (n = 15)	-0.14	-4.83	2.99	-2.19
**Freeze and thaw cycles (RSE%)**				
LQC (n = 4)	-5.42	3.83	-6.46	-3.42
HQC (n = 4)	-0.43	-5.35	-0.99	9.61
**Short-term (RSE%)**				
LQC (n = 4)	-3.25	-3.08	-10.00	-5.38
HQC (n = 4)	-1.19	-5.27	3.18	7.77
**Post-preparative (RSE%)**				
LQC (n = 4)	-6.00	3.83	4.17	-3.29
HQC (n = 4)	-1.87	-13.79	4.11	-4.05

Figure S1. Chromatograms of blank plasma samples. Peaks: C1- mexiletine (IS); C2- endoxifen; C3- 4-hydroxy-tamoxifen; C4- N-desmethyltamoxifen; C5- tamoxifen.

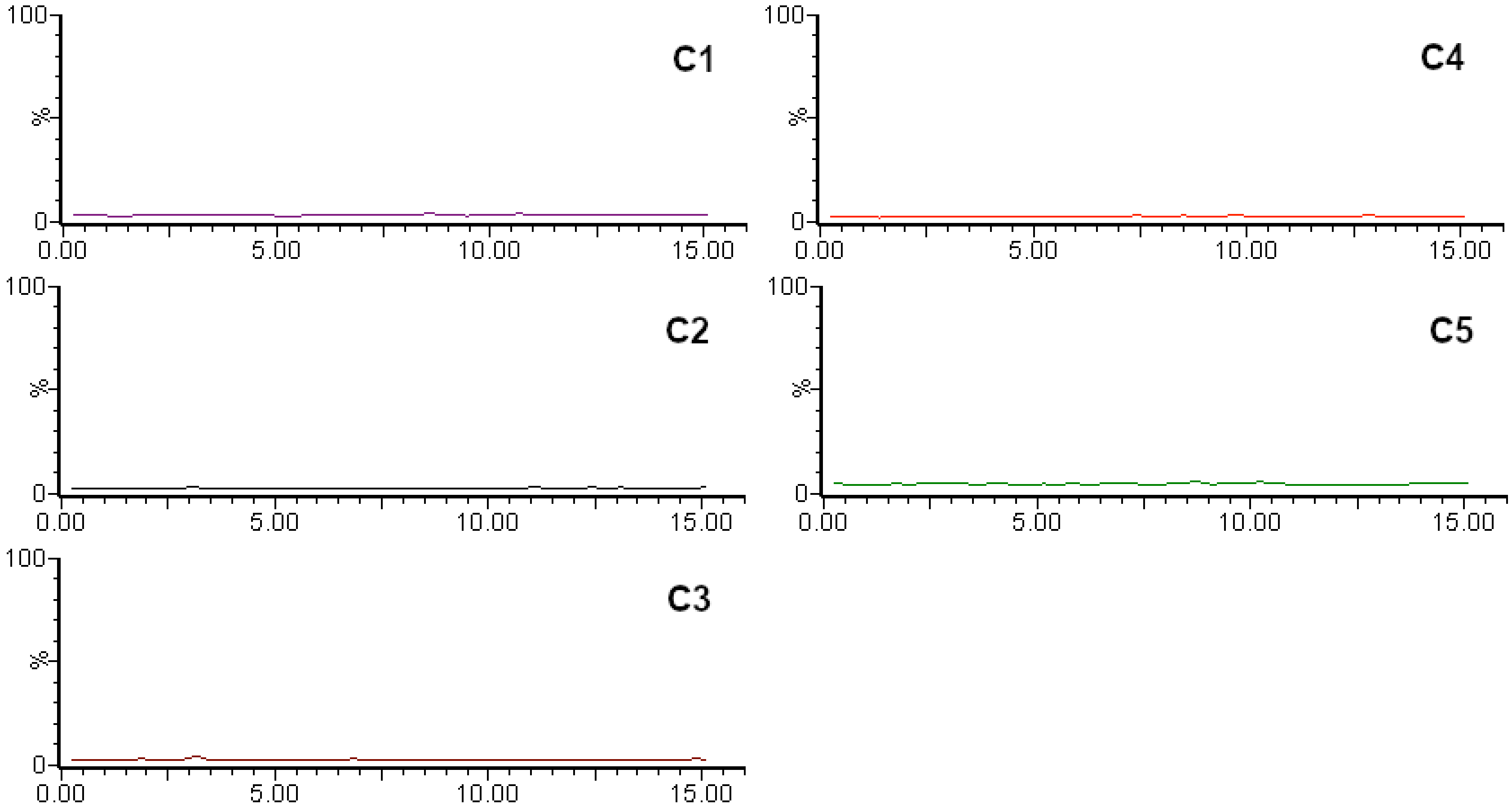

Figure S2. Mass spectra of protonated tamoxifen (A1) and its product F1 (A2); 372.6 > 71.9 m/z.

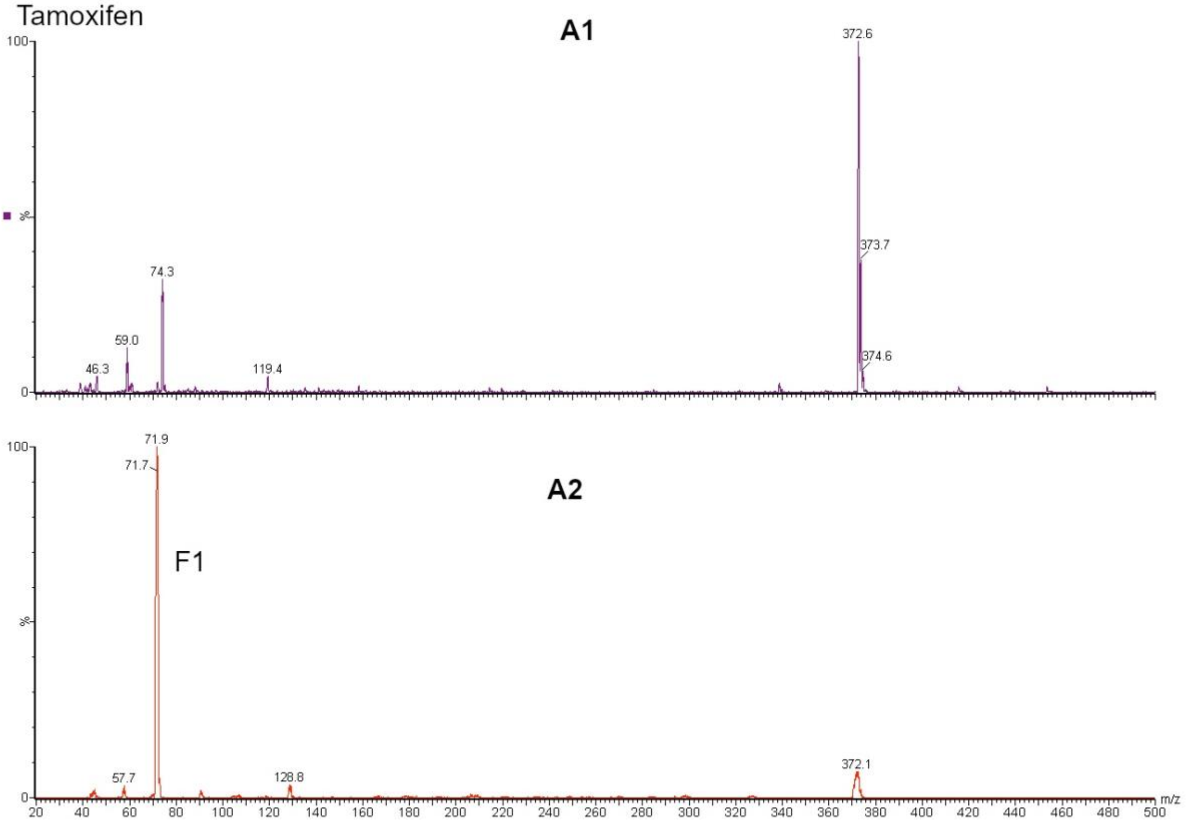

Figure S3. Mass spectra of protonated endoxifen (B1) and its product F2 (B2); 374.96 > 57.8 m/z.

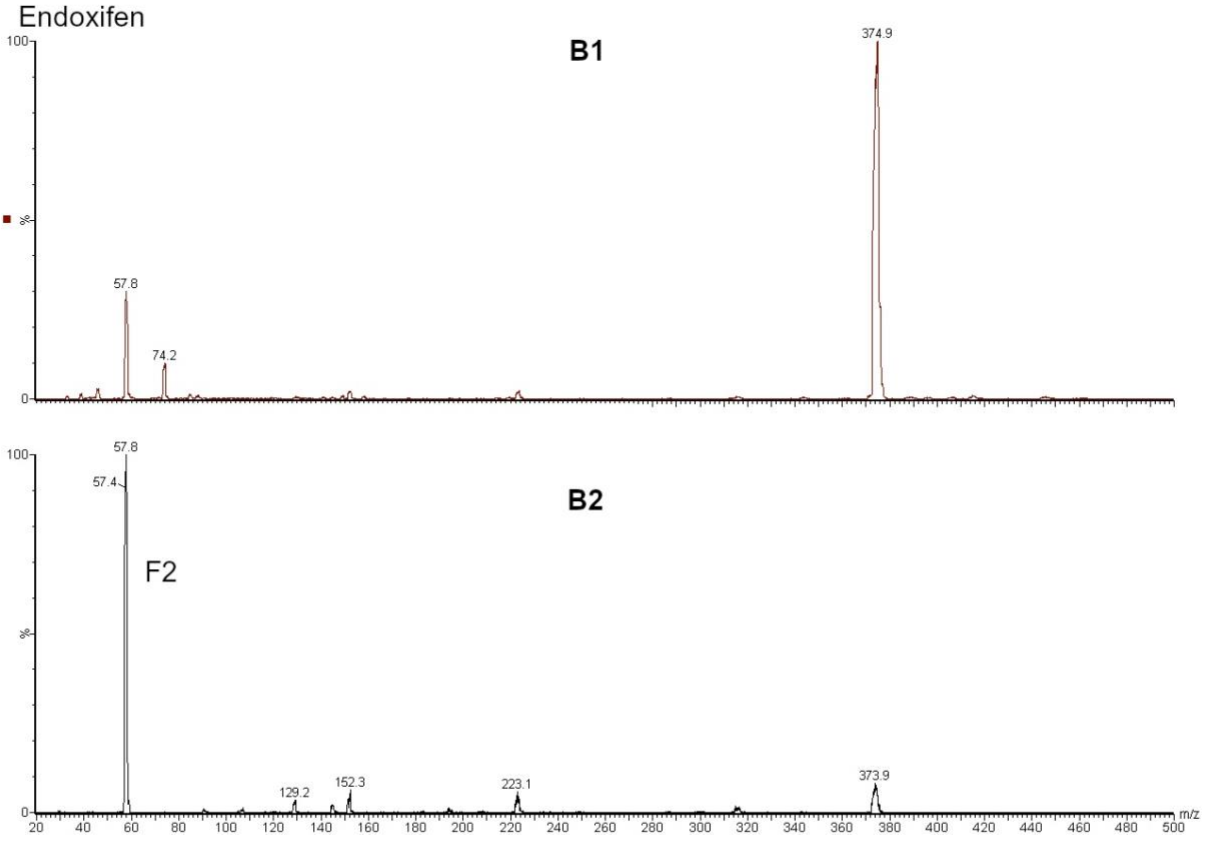

Figure S4. Mass spectra of protonated 4-hydroxy-tamoxifen (C1) and its product F3 (C2); 388.7 > 71.9 m/z.

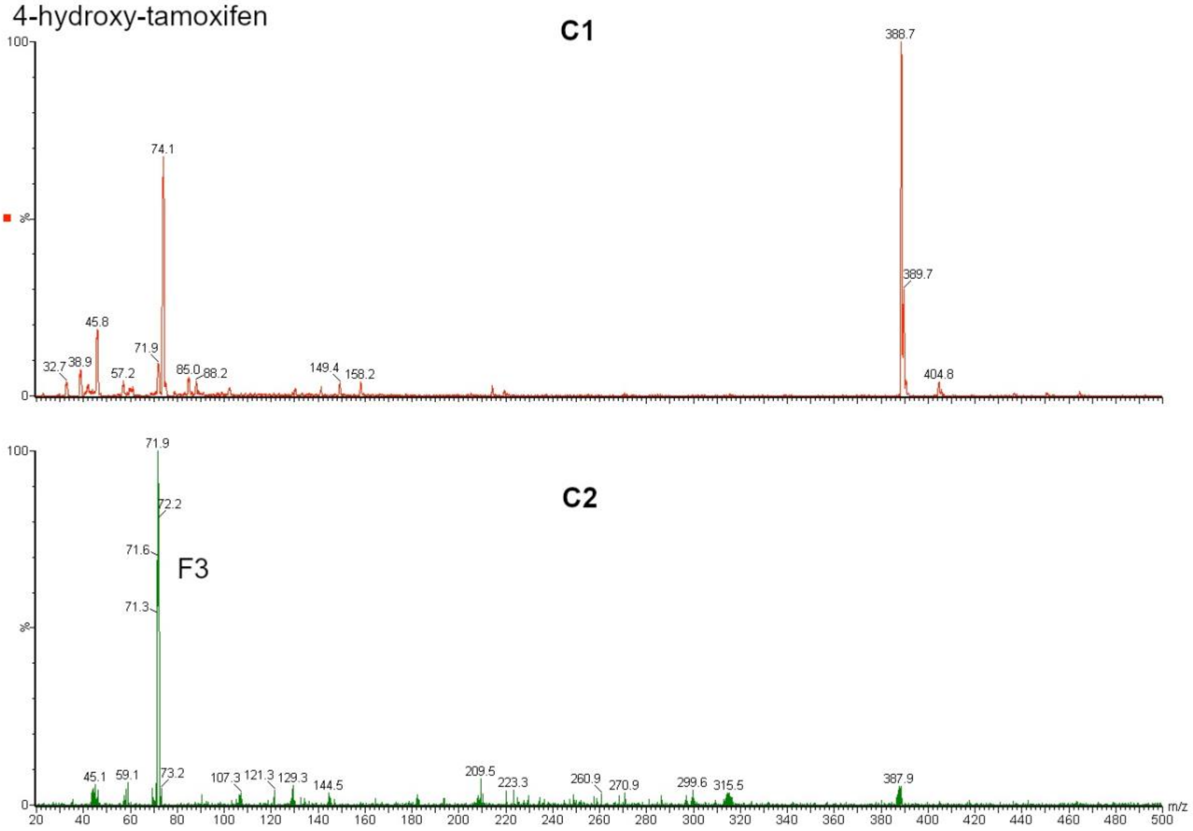

Figure S5. Mass spectra of protonated N-desmethyltamoxifen (D1) and its product F4 (D2); 358.9 > 57.5 m/z.

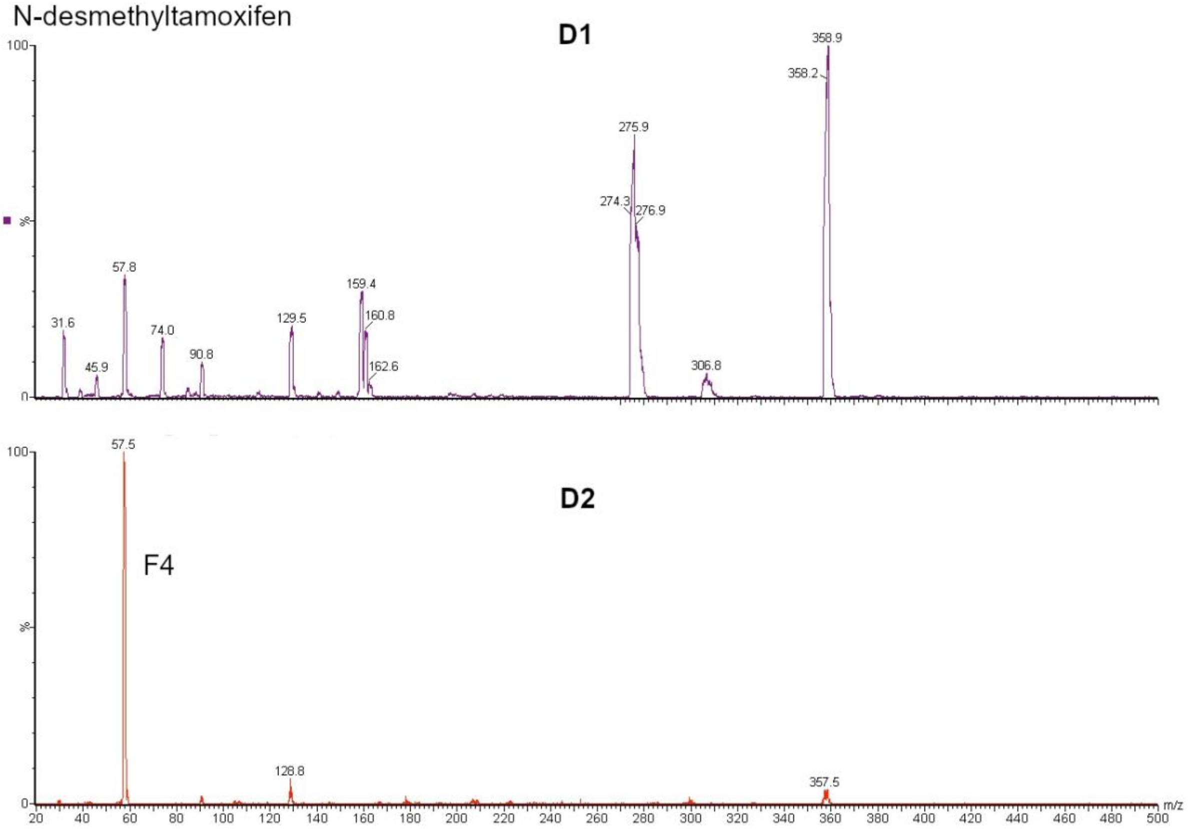

Figure S6. Mass spectra of protonated mexiletine (IS) (E1) and its product F5 (E2); 180 > 58 m/z.

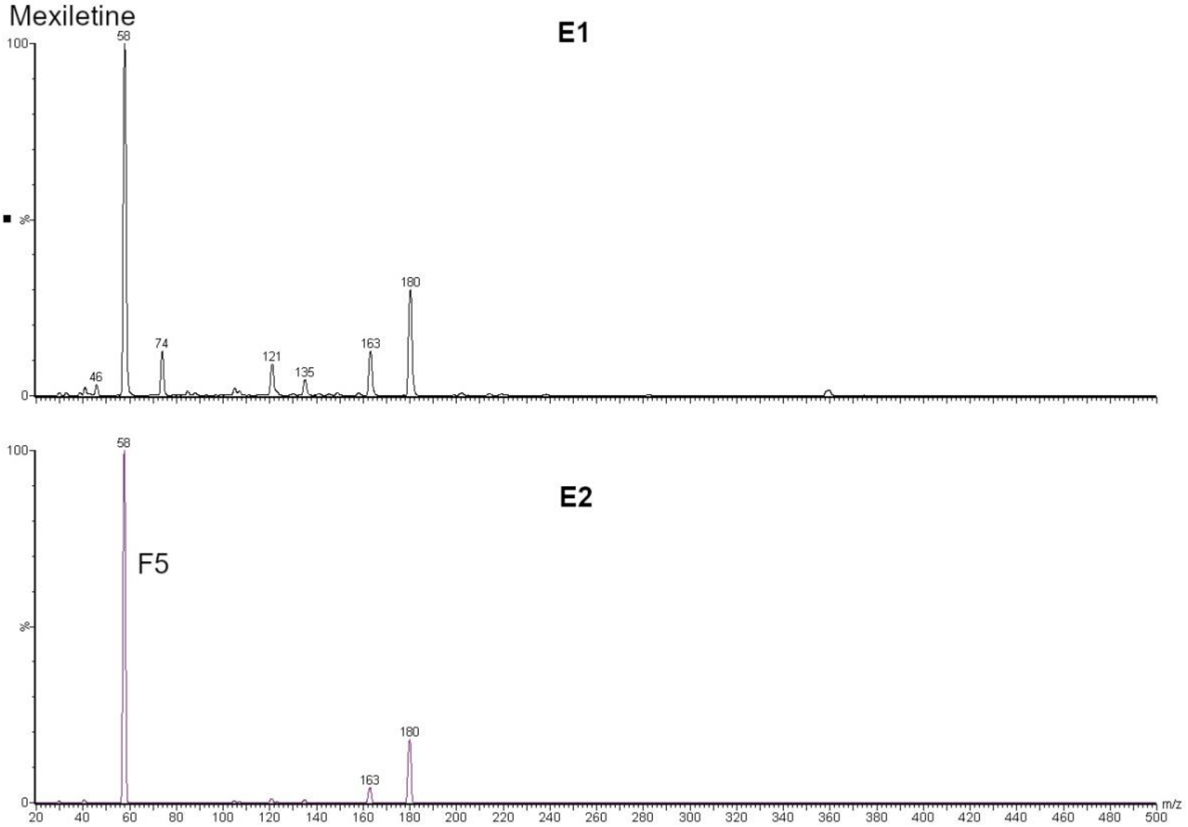

Figure S7. Chromatograms of plasma samples obtained at 1.5h after administration of 20mg tamoxifen dose (A1-A5). Chromatograms of blank plasma samples added of 5 ng/mL of tamoxifen, endoxifen, 4-hydroxy-tamoxifen, N-desmethyltamoxifen (B1-B5). Peaks: 1-mexiletine (IS); 2- endoxifen; 3- 4-hydroxy-tamoxifen; 4- N-desmethyltamoxifen; 5-tamoxifen.

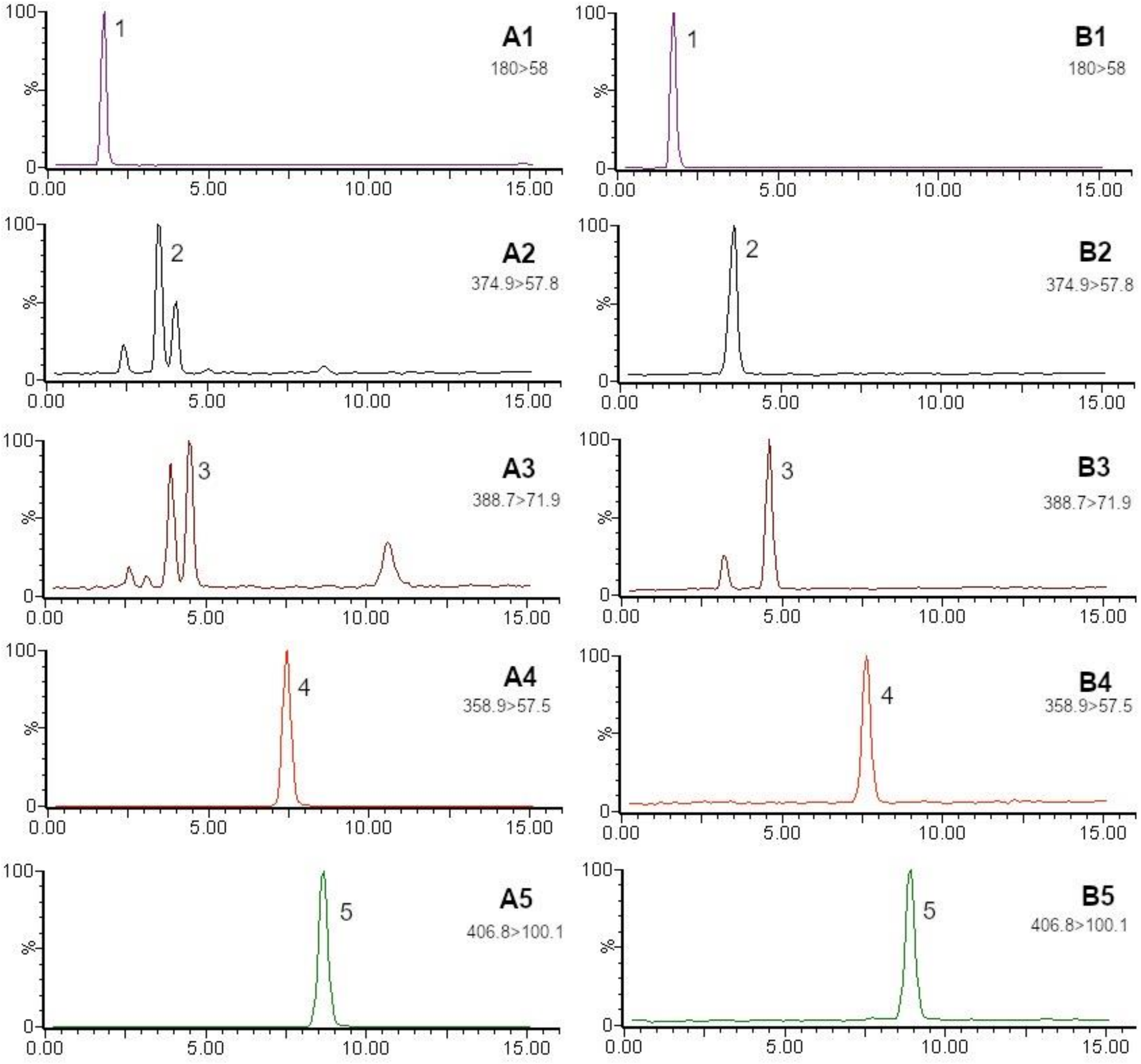

3. References

1. Food and Drug Administration (FDA), Bioanalytical Method Validation: Guidance for Industry. 2018. https://www.fda.gov/files/drugs/published/Bioanalytical-Method-Validation-Guidance-for-Industry.pdf

2. European Medicines Agency (EMA). Guideline on bioanalytical method validation. 2011. https://www.ema.europa.eu/en/documents/scientific-guideline/guideline-bioanalytical-method-validation_en.pdf
